# EmAtlas: a comprehensive atlas for exploring spatiotemporal activation in mammalian embryogenesis

**DOI:** 10.1093/nar/gkac848

**Published:** 2022-10-03

**Authors:** Lei Zheng, Pengfei Liang, Chunshen Long, Haicheng Li, Hanshuang Li, Yuchao Liang, Xiang He, Qilemuge Xi, Yongqiang Xing, Yongchun Zuo

**Affiliations:** State Key Laboratory of Reproductive Regulation and Breeding of Grassland Livestock, School of Life Sciences, Inner Mongolia University, Hohhot, 010070, China; State Key Laboratory of Reproductive Regulation and Breeding of Grassland Livestock, School of Life Sciences, Inner Mongolia University, Hohhot, 010070, China; State Key Laboratory of Reproductive Regulation and Breeding of Grassland Livestock, School of Life Sciences, Inner Mongolia University, Hohhot, 010070, China; State Key Laboratory of Reproductive Regulation and Breeding of Grassland Livestock, School of Life Sciences, Inner Mongolia University, Hohhot, 010070, China; State Key Laboratory of Reproductive Regulation and Breeding of Grassland Livestock, School of Life Sciences, Inner Mongolia University, Hohhot, 010070, China; State Key Laboratory of Reproductive Regulation and Breeding of Grassland Livestock, School of Life Sciences, Inner Mongolia University, Hohhot, 010070, China; State Key Laboratory of Reproductive Regulation and Breeding of Grassland Livestock, School of Life Sciences, Inner Mongolia University, Hohhot, 010070, China; State Key Laboratory of Reproductive Regulation and Breeding of Grassland Livestock, School of Life Sciences, Inner Mongolia University, Hohhot, 010070, China; The Inner Mongolia Key Laboratory of Functional Genome Bioinformatics, School of Life Science and Technology, Inner Mongolia University of Science and Technology, Baotou, 014010, China; State Key Laboratory of Reproductive Regulation and Breeding of Grassland Livestock, School of Life Sciences, Inner Mongolia University, Hohhot, 010070, China

## Abstract

The emerging importance of embryonic development research rapidly increases the volume for a professional resource related to multi-omics data. However, the lack of global embryogenesis repository and systematic analysis tools limits the preceding in stem cell research, human congenital diseases and assisted reproduction. Here, we developed the EmAtlas, which collects the most comprehensive multi-omics data and provides multi-scale tools to explore spatiotemporal activation during mammalian embryogenesis. EmAtlas contains data on multiple types of gene expression, chromatin accessibility, DNA methylation, nucleosome occupancy, histone modifications, and transcription factors, which displays the complete spatiotemporal landscape in mouse and human across several time points, involving gametogenesis, preimplantation, even fetus and neonate, and each tissue involves various cell types. To characterize signatures involved in the tissue, cell, genome, gene and protein levels during mammalian embryogenesis, analysis tools on these five scales were developed. Additionally, we proposed EmRanger to deliver extensive development-related biological background annotations. Users can utilize these tools to analyze, browse, visualize, and download data owing to the user-friendly interface. EmAtlas is freely accessible at http://bioinfor.imu.edu.cn/ematlas.

## INTRODUCTION

Early mammalian development encompasses several critical timing events, including nuclear reprogramming, zygotic genome activation, cell lineage differentiation, and organogenesis (1–3). The advent of next-generation sequencing technologies and rapid data generation for multi-omics (4–6) have considerably broadened our understanding of reconstructing the epigenetic landscape, global triggering of transcription waves, cell fate decision and the routines of gene regulatory networks during embryo development (3,7–9). However, most researches on early mammalian development concentrate on a single tissue and the multi-omics data are scatted across different repositories, and it is also still a ‘black box’ that how each tissue completes cell specialization through spatiotemporally dynamic gene expression under the guidance of the blueprint from the zygote genome (10).

Several databases have been developed for deciphering early mammalian embryogenesis (11–16). For instance, DBTMEE and EMAGE integrate gene expression data to investigate developmental patterns in mouse early embryos (11,13). BGDB analyses the functional diversity and regulatory roles of 6897 bivalent genes in human and mouse embryo stem cells (ESCs) (12). DevMouse comprises a variety of analytic tools for quantifying mouse developmental methylomes (14). EmExplorer features transcriptome data from five species concerning temporal activation of gene expression and 306 development-related pathways (15). DevOmics stores multi-omics data from human and mouse early embryos and offers several downstream analysis tools, such as differentially expressed genes analysis and data visualization (16). The human cell landscape (HCL) and the mouse cell atlas (MCA) constructed by Guo *et al.* (9,17) offer a vital resource for the study of developmental biology. These databases and datasets promote the proper understanding of the basic embryogenesis mechanisms. However, they paid more attention to the local embryogenesis path and the cell identity is poorly given presented, which will hinder our process to decode the regulatory mechanism of embryo development (Table 1). Therefore, it is extremely desirable to construct a global and comprehensive database that covers the complete spatiotemporal development landscape in mammalian embryogenesis and offers diverse analysis tools for identifying cell identity and biomarkers on different omics levels.

**Table 1.** Overview of several key aspects of similar databases

Database	Development stage	Omics	Analysis tools
EmAtlas	Oocyte	Gene expression	EmRanger Enrichment Tools
	Sperm	Chromatin accessibility	Development Genome Browser,
	Preimplantation embryo	DNA methylation	Development Tissue/Cell Biomarker Analysis
	Postimplantation embryo	Nucleosome occupancy	Spatiotemporal Expression Analysis
	Fetal and neonatal (65 tissues)	HMs (28 types)	Spatiotemporal Biological Pathway Analysis
	ESCs	TFs (53 types)	Development Disease/Drug Analysis
			Embryo Mutil-Omics Atlas
DevOmics	Preimplantation embryo	Gene expression	Differentially Expressed Gene Analysis
		Chromatin accessibility	Genome Browser
		DNA methylation	
		HMs (4 types)	
BGDB	ESCs	HMs (2 types)	No
DBTMEE	Preimplantation embryo	Gene expression	Genome Browser
			Gene Network
EmExplorer	Preimplantation embryo	Gene expression	Pathway Analysis
DevMouse	Preimplantation embryo	DNA methylation	MethyBrowser
	Postimplantation embryo		
EMAGE	Preimplantation embryo	Gene expression	IIP3D Viewser
	Postimplantation embryo		

Here, we developed the EmAtlas, which collects the most comprehensive multi-omics data on mammalian development and is committed to exploring spatiotemporal activation of embryogenesis. The professional resource involved spatiotemporal landscape in mouse and human across time points from gametogenesis to fetal. It is mainly committed to discovering novel potential tissue biomarkers, analyzing cell heterogeneity, browsing the spatiotemporal epigenetic profiles and biological pathway, and exploring the functional domains and structures of pioneer protein factors. EmRanger was first proposed to annotate biomarkers more extensively during temporal development.

## MATERIALS AND METHODS

### Data curation and integration

**Figure 1.**
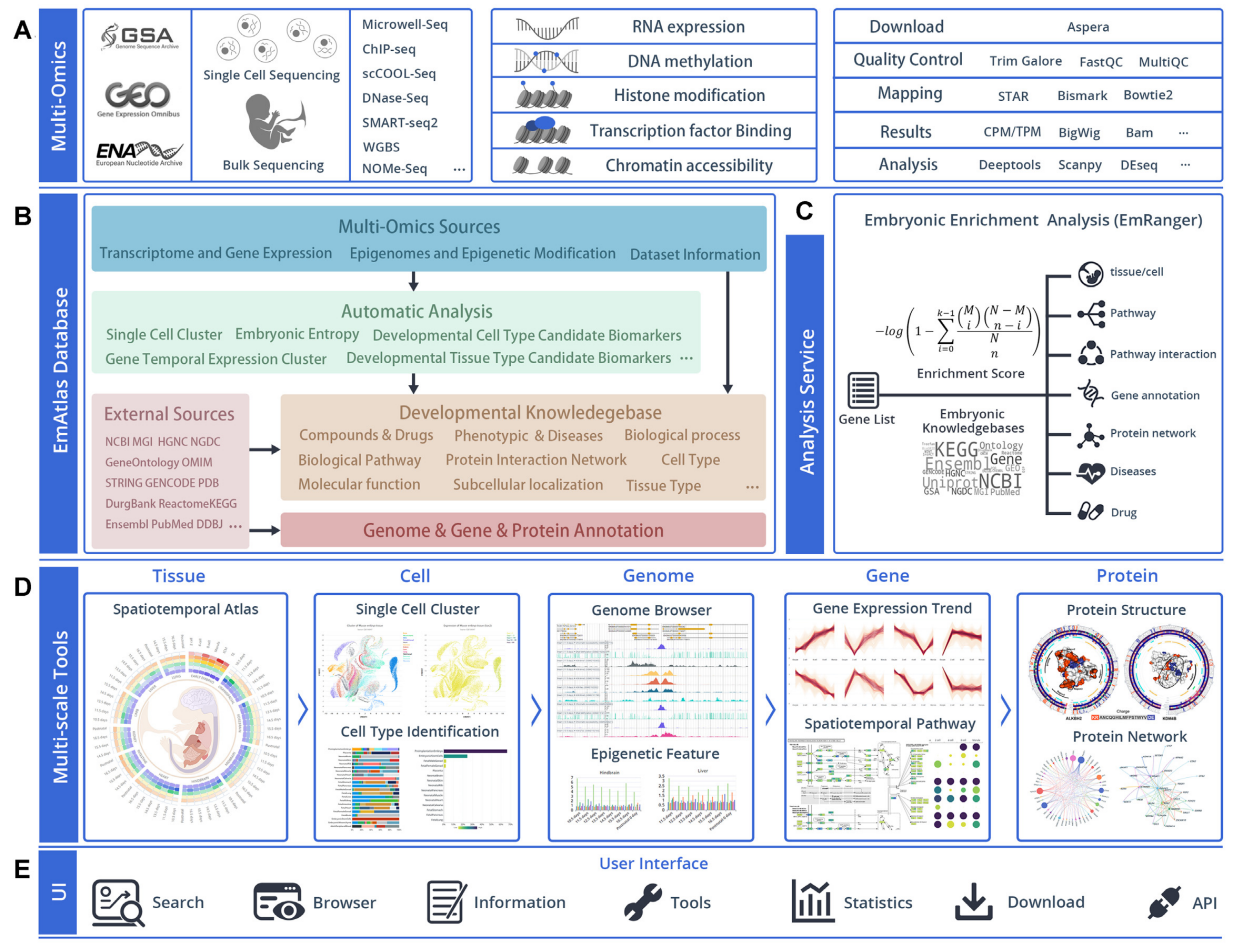
The framework of the EmAtlas. (**A**) Collection of the datasets and design of pipeline for each omics. (**B**) Construction of multi-omics resources and development knowledgebase. (**C**) The workflow of the EmRanger analysis service. (**D**) The tools of five scales. (**E**) The user interface.

The framework of the EmAtlas is described in Figure 1. We collected datasets related to mammalian embryogenesis from Gene Expression Omnibus (GEO), European Nucleotide Archive (ENA) and Genome Sequence Archive (GSA). 9113 samples from 821 multi-omics datasets involving the most popular sequencing technologies were integrated into this resource. It was archived according to the spatiotemporal development of preimplantation embryos, fetuses, and neonates, with 65 tissues and 116 cell types included. The normalized data were then processed through a unified pipeline to obtain single-cell clusters, gene expression trend clusters, embryonic development entropy, highly variable genes, modification signal values, and so on. In addition, the development annotations of 23 authoritative knowledge bases were reviewed and integrated into the database. We also mined large-scale data and knowledgebases and obtained candidate biomarkers in developmental tissue/cell types. Furthermore, we developed EmRanger, an enrichment system focused on development-related gene sets with five levels of analysis tools (tissue, cell, genome, gene, and protein).

### Implementation

We used cloud platform technology to develop EmAtlas and adopted a framework separating the database, front-end and back-end, which were distributed and deployed on three servers. This strategy enhances the security, scalability, and concurrency of EmAtlas. The pipeline of data pre-processing and analysis can be found in the Figure S1. A total of 64 TB of data was stored in MongoDB (for redundant data) and MySQL (for curated data). The sharding strategy was implemented to improve query and management efficiency. The back-end was hosted on a high-performance computing server, which used Nginx as HTTP. The API was implemented in Python using the Django framework. For the EmRanger service, after the user uploads the gene list to the server and sets essential parameters, EmAtlas temporarily stores the inputs and creates a job with a unique ID. The job is subsequently submitted into a task queue, where it will be handled asynchronously and allocated sufficient computing resources. Once the job is processed, the frontend will generate the analytical report.

The user interaction was developed using Vue.js, designed for modern browsers to create a dynamically rendered webserver. The graphic viewers were implemented utilizing D3.js, echart.js, and highchart.js. The user requests from the web interface are sent to Nginx via RESTful API to communicate with the backend server.

## RESULTS

### Overview of EmAtlas

The EmAtlas presents the complete spatiotemporal landscape in mouse and human, from gametogenesis to preimplantation, and eventually to fetus and neonate (Table 1). The database contains multi-omics data, involving gene expression, chromatin accessibility, DNA methylation, nucleosome occupancy, 28 types of histone modifications (HMs), and 53 transcription factors (TFs). The analysis tools and EmRanger module enable flexible exploration of associations between molecular events of interest and specific embryogenesis processes, providing the opportunity to identify underlying regulators of embryogenesis. The user-friendly interface allows users to analyse, browse, visualize, and download data/images.

### Tools of five scales in EmAtlas

EmAtlas provides analysis tools to describe five scales of mammalian embryogenesis, including tissue, cell, genome, gene and protein, and allows users to explore development-associated entropy, biological pathway, interaction network and cell/tissue biomarkers within different stages.

#### At the tissue level

**Figure 2.**
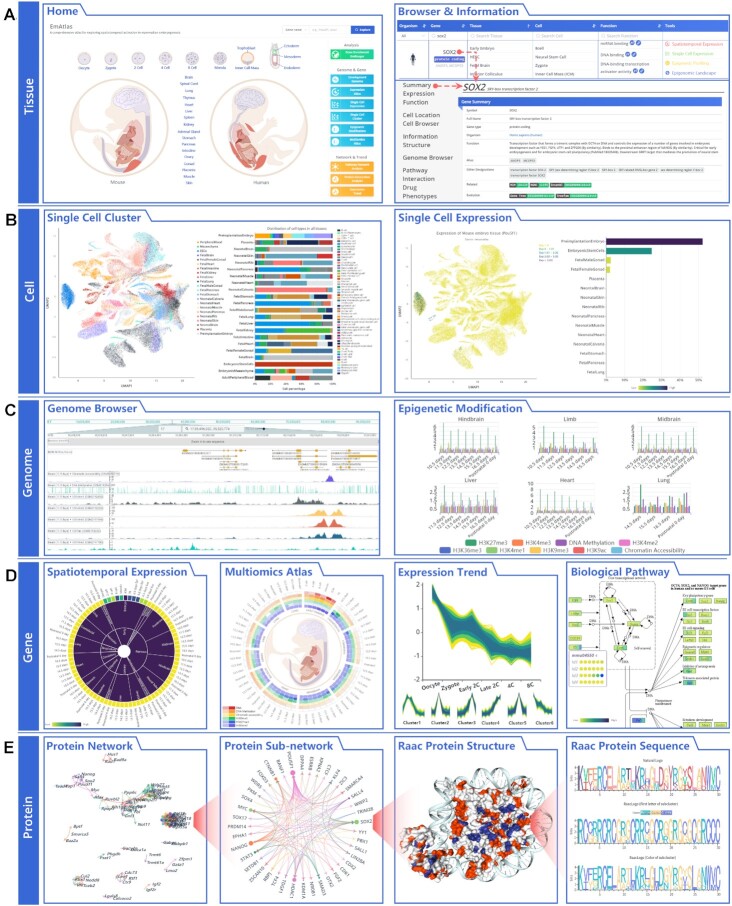
Multi-level analysis tools in EmAtlas. (**A**) At the tissue level, the Home provides a viewer across development stages in tissues and shortcut cards for analysis tools. The browser tool shows tissue-associated gene annotation and analysis tools. (**B**) At the cell level, the platform contains a Single Cell Cluster and Single Cell Expression tool. (**C**) At the genome level, Genome Browser and Epigenetic Modification were developed to decipher the spatiotemporal genome modification states. (**D**) At the gene level, Spatiotemporal Expression, Multiomics Atlas, Expression Trend, and Biological Pathway tools were provided to explore the expression patterns of genes. (**E**) At the protein level, protein interaction networks were constructed, and RaacFold and RaacLogo tools were integrated to explore essential proteins in development.

The Home page displays the early embryonic and tissue atlas (Figure 2A). Users can click on the tissue name to browse the candidate biomarkers of tissue, and biological pathways, and further explore spatiotemporal expression, single-cell expression, epigenetic modification score, and epigenomic profile. In the browser tool, we provide an independent fuzzy search component for each annotation field. In the tissue field, users can enter a tissue name to extract the related results. While in the gene field, users are allowed to directly use gene aliases to extract the results of gene symbols. For example, *ANOP3* can be used to obtain the tissue type that specifically expresses *SOX2*. In this way, candidate biomarkers can be annotated from the perspective of phenotype, disease, drug, compound and other information.

#### At the cell level

EmAtlas can cluster single cells into different cell groups based on gene expression, and the identified potential cellular biomarkers, which provides a global map of cell populations (Figure 2B). The global cell map can be obtained from the Single Cell Cluster card on the home page. This tool provides UMAP and TSNE visualizations to show cell heterogeneity, which can distinguish cells according to tissue type, cell type, and the cluster results of the Leiden algorithm. In addition to the global cell map, users can choose interested tissue to view its sub-cluster map to explore more detailed cell types and their proportions. To further delineate gene expression in different tissues/cells, we developed a single-cell expression pattern analysis tool. The Single Cell Expression card on the home page can be used to identify tissues and cells that specifically express a gene in the view. To quantify the above observations, we provided the proportion and number of cells expressing a specific gene in each cell population. Users can set two parameters as needed to draw custom single-cell expression profiles.

#### At the genome level

To decipher the spatiotemporal epigenetic modification states of mammalian embryogenesis at a single-base resolution, EmAtlas provides a genome browser including chromatin accessibility, DNA methylation, HMs, and TFs (Figure 2C). It exhibits the information of data source, tissue type, development stage and omics data type. Users can view patterns of multi-omics in the various tissues across the developmental course and infer biological conclusions. EmAtlas also offers an Epigenetic Modification Analysis Tool to quantify the epigenetic signals in different gene regions, such as promoter, untranslated region (UTR) and coding sequence (CDS). These signals are described by three values: modification signal area, single base modification value, and modification coverage percentage.

#### At the gene level

Firstly, to explore the gene expression patterns during mammalian development, we interpreted them as three layers of the body system, tissue, and development stage that are gradually deepened, and showed them in the three rings of the sunburst diagram, respectively, with gene expression values at the outermost (Figure 2D). The analysis tool of spatiotemporal gene expression was developed, allowing users to view the specific gene expression in a system, tissue, or stage. In addition, it enables users to view the signal strength of multi-omics for a gene in the temporal development of various tissues. The tool supports NCBI, Ensembl identifier and aliases search for genes, transcripts and proteins. Secondly, deciphering the temporal expression patterns of the gene is a crucial step toward understanding the regulation mechanisms of embryogenesis, so we designed a trend analysis tool based on the Fuzzy C-Means algorithm. It is possible to obtain gene sets with similar expression trends with this tool, and it is worth mentioning that the tool can employ the genetic evolution algorithm to filter the gene set and define whether it is old or young. Finally, orderly regulation is inseparable from biological pathways, and pathways do not show the time points of development. So, we developed a biological pathway map tool that can view the time trajectories of different tissues. All transcripts in the same pathway are plotted in a bubble plot and can be screened according to expression abundance and percentage of cells.

#### At the protein level

Analysing the interaction network between proteins, and the sequence and structural basis of protein function is a necessary step in studying the healthy development of mammals. We built a global protein interaction network based on tissue candidate biomarkers and allowed users to customize the protein sub-regulation network according to tissue types and essential genes (Figure 2E). The tool also supports different gene id searches. EmAtlas integrated RaacLogo (18) and RaacFold (19) tools to explore key protein sequences, functional domains, and structures in embryonic development. Both can make the sequence/structure more concise and clearer to reflect protein conservation, mutation sites, homology sequences, and biological functions.

### EmRanger

It has become a standard practice to employ multi-omics analysis to deconstruct the molecular mechanisms during mammalian embryogenesis. In this process, several candidate gene sets with dramatic changes in expression and epigenetic modifications are usually identified. However, the function, biological descriptions and regulatory relationship of these candidate genes remain poorly understood. Compared with the popular enrichment analysis tools (such as DAVID, clusterProfiler, Metascape and Enrichr), the advantage of EmRanger can be summarized as follows: (i) By Manual correction, 23 functional knowledge bases were incorporated into the specialized annotation library for EmRanger. (ii) The professional annotation of categories of tissue, cell, phenotype, and drug information were presented. (iii) The crosstalk between pathways and interactions between proteins in different tissues are integrated into the result reports. So, EmRanger can obtain a more comprehensive and specialized analysis report in multiple levels for development biology.

EmRanger involves ten aspects including development-related tissues, cell types, biological pathways, pathway networks, phenotypic diseases, drug compounds, subcellular localization, biological processes, molecular functions and protein interactions. The analysis system adopts CAME (Conversion, Annotation, Membership, Enrichment) to build a workflow, where Conversion is used to convert the gene identifiers in the input gene list to other identifiers including Ensemble ID, Entrez ID; Annotation provides the basic annotation information in the gene list, such as gene name, function description and gene type; Membership classifies user-uploaded gene sets with different biological keyword similarities, such as sub-gene sets related to stem cell reprogramming. In the enrichment analysis system, the significance evaluation score for the biological description involved in the uploaded gene set is calculated using hypergeometric distribution:

(1) $$\begin{equation*}score = - log\left( {1 - \mathop \sum \limits_{i = 0}^{k - 1} \frac{{\left( {\begin{array}{@{}*{1}{c}@{}} M\\ i \end{array}} \right)\left( {\begin{array}{@{}*{1}{c}@{}} {N - M}\\ {n - i} \end{array}} \right)}}{{\begin{array}{@{}*{1}{c}@{}} N\\ n \end{array}}}} \right)\end{equation*}$$

**Figure 3.**
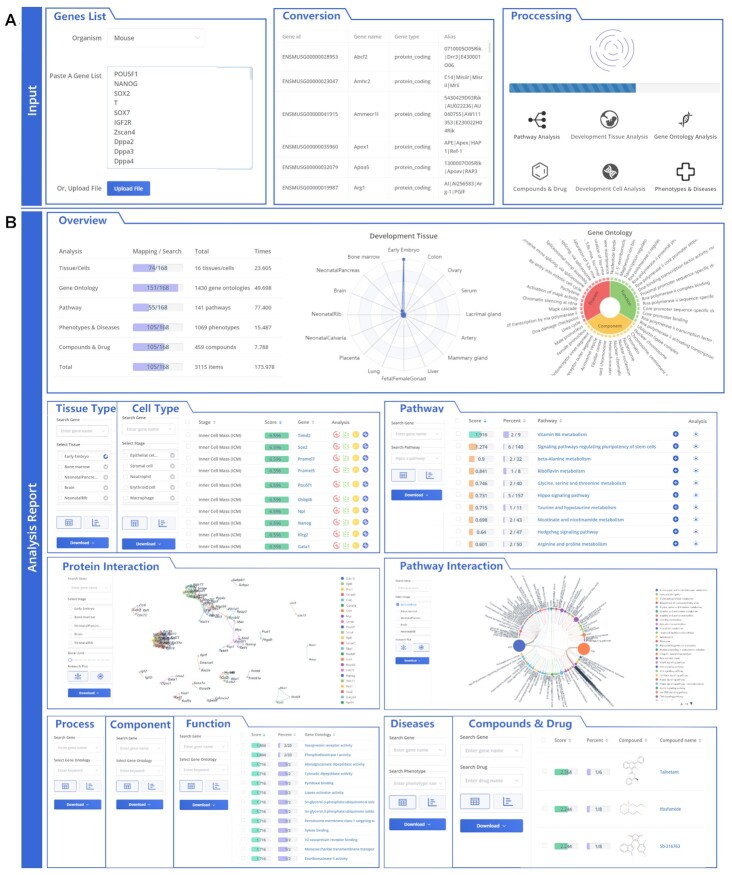
The workflow of EmRanger. (**A**) Input genes list for enrichment. (**B**) The EmRanger analysis report including many aspects, such as tissue type, cell type, biological pathway, pathway interaction, biological process, subcellular localization, molecular function, protein interaction, phenotype & disease and drug & compound.

Among them, *N* is the total number of genes involved in a certain type of functional annotation library; *M* is the total number of genes involved in a description in the library; *n* is the number of genes in the gene set uploaded by the user; *k* is the number of *n* belonging to *M*. The evaluation scores of biological descriptions are divided into four grades: extremely significant (score > 3), very significant (2 ≤ score < 3), significant (1.3 ≤ score < 2), and not significant (score < 1.3). Figure 3 shows an overview of the input, analysis, and visualization. The detailed description of using EmRanger for gene enrichment to obtain multi-faceted developmental annotations was presented as follows.

#### Input gene list

Users can visit EmRanger (http://bioinfor.imu.edu.cn/ematlas/#/emranger), and type or paste a list containing one or several gene names in the textbox (Figure 3A). Alternatively, it is possible to upload a gene list file. Then, Genes are detected and annotated with their Ensembl ID, genotype, and other information. Finally, the above data will be submitted to the backend server for analysis.

#### Analysis report

After the analysis is completed, an online analysis report will be generated. The green, blue, orange, and red are used to distinguish different significance scores (Figure 3B). The first panel is the overview of the enrichment information of the gene list uploaded by the user. The statistics table sequentially displays the gene match rate, the number of captures, and the analysis time. The radar map in the middle shows the top 20 developmental tissues based on EmRanger.

The following pie chart shows the Gene Ontology enrichment results of user-uploaded gene lists. At the bottom of the analysis report, there are pathways, phenotypes, and drug information. For more detailed information, users can click the panel of each enrichment category at the top. Besides, each analysis panel supports dynamic filtering and downloading.

### APPLICATION

**Figure 4.**
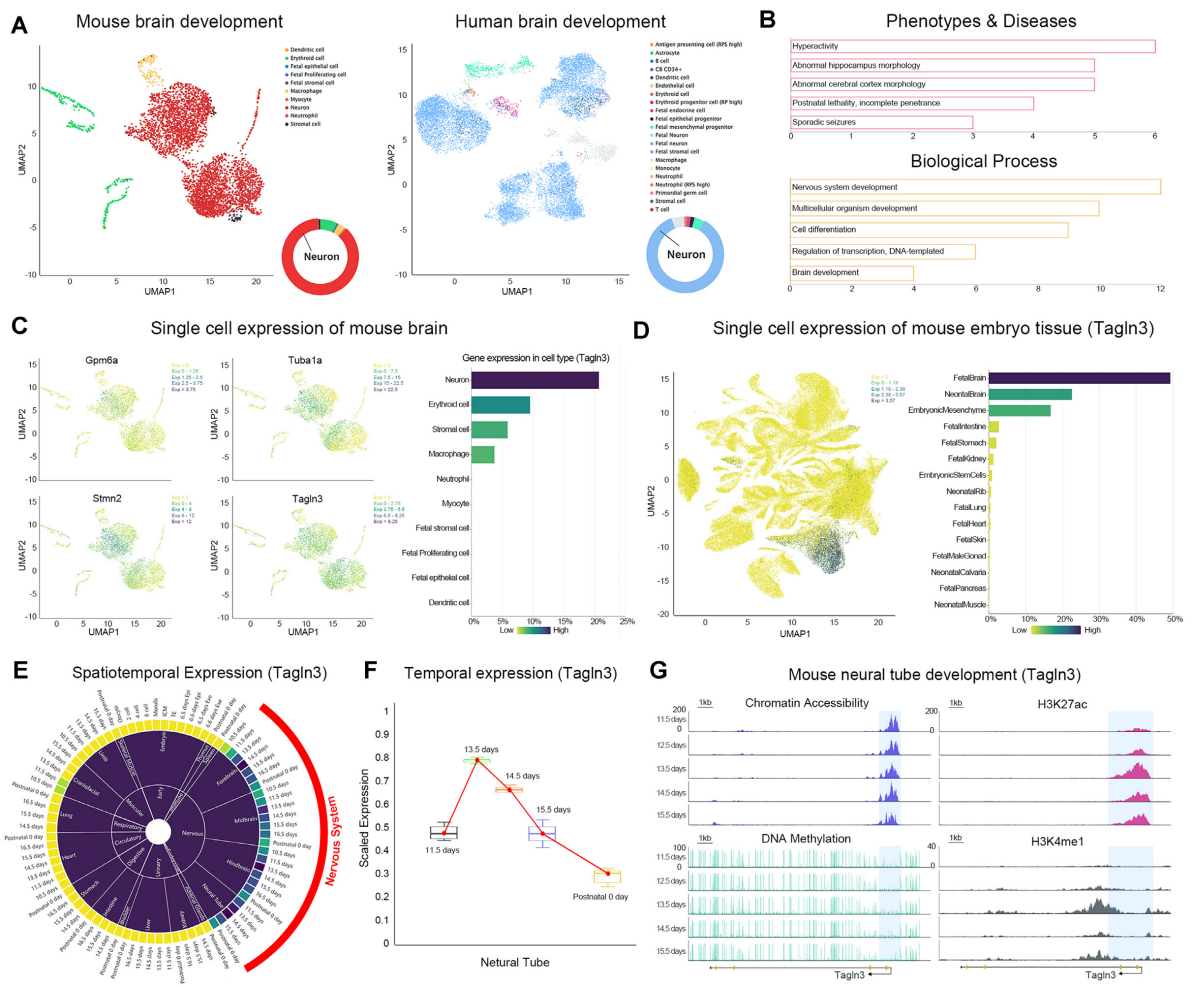
EmAtlas predicts potential regulators of tissue development that are conserved cross-species. (**A**) Uniform Manifold Approximation and Projection (UMAP) of 4369 individual cells in mouse fetal brain and 13625 individual cells in human fetal brain, colored by cell types. (**B**) Enrichment analysis of neuron-specific HVGs shared by human and mouse based on EmRanger. (**C**) UMAP plot displaying the expression level of *Gpm6a*, *Tuba1a*, *Stmn2*, and *Tagln3* as well as the proportion of cells expressing *Tagln3* in the different brain cells. (**D**) Global expression profile of *Tagln3* in single cells of all tissues during mouse embryogenesis. Gene expression levels are indicated by different colors. And the proportion of cells expressing *Tagln3* in different tissues was shown behind. (**E**) The spatiotemporal expression profile of *Tagln3* during mouse embryogenesis. (**F**) The temporal expression pattern of *Tagln3* during mouse neural tube development. (**G**) The genome browser views showing chromatin accessibility, H3K27ac, DNA methylation, and H3K4me1 enrichment during mouse neural tube development near *Tagln3*.

A typical application of EmAtlas is exploring potential regulators of tissue development that are conserved cross-species. Take the development of the fetal brain as an example. The Single Cell Cluster tool exhibited that neurons are the main cell type in human and mouse fetal brains, with a percentage exceeding 80% (Figure 4A). Thus, we focused on neuron development and identified neuron-specific high variation genes (HVGs) shared by human and mouse. EmRanger analysis results showed that these HVGs are mainly enriched in some biological processes related to the nervous system and brain development (Figure 4B). And the dysregulation of these HVGs can lead to the occurrence of diseases such as hyperactivity, sporadic seizures, and abnormal brain morphogenesis. The above analysis implied that there may be a similar mechanism of neuron development between human and mouse, suggesting the feasibility of using the mouse as a model animal for studying human neurodevelopmental diseases. Subsequently, four representative genes (*Stmn2*, *Tuba1a*, *Gpm6a* and *Tagln3*) specifically expressed in the neurons were selected (Figure 4C). *Stmn2*, *Tuba1a* and *Gpm6a* have been confirmed to play crucial roles in early neurogenesis (20,21) and the maintenance of neuronal morphology (22,23).

*Tagln3* was considered as a pan-neuronal marker in the previous studies (9,24), but the spatiotemporal expression pattern and dynamic epigenomic landscape of *Tagln3* during neurogenesis remain poorly characterized. EmAtlas further proved *Tagln3* was specifically expressed in mouse brain tissue, including the forebrain, midbrain, hindbrain, and neural tube, with the neural tube having the highest expression level (Figure 4D, E). Notably, the *Tagln3* reached maximal expression levels in the fetal neural tube on day 13.5, and then gradually declined until the postnatal stage (Figure 4F). Moreover, high chromatin accessibility in the *Tagln3* promoter was parallel to the deposition of H3K27ac and H3K4me1, and low methylation levels from day 11.5 to day 15.5, displaying distinct epigenomic dynamics during neural tube development (Figure 4G). Of note, H3K27ac was markedly enriched in the neural tube on day 13.5, coinciding with the high expression level of *Tagln3* in the neural tube on day 13.5. These results revealed that *Tagln3* may serve as a critical regulator to shape the early development of the nervous system. Collectively, EmAtlas provides resources and theoretical guidance for experimental biologists to identify potential regulators and decipher their molecular mechanisms in cell fate decisions during embryogenesis.

## DISCUSSION

The emergence of single-cell multi-omics sequencing technologies enables us to investigate embryo heterogeneity and cell fate decision with unprecedented resolution. The construction of the database embracing a huge amount of sequencing data has become increasingly important in revealing the regulatory mechanism of embryogenesis. Compared to existing databases, EmAtlas presents a more comprehensive multi-omics resource of mammalian embryogenesis and versatile multi-scale tools serving as different omics layers with personalized solutions. EmAtlas provides a user-friendly interface to query, browse, analyse and visualize multi-omics data. Multi-scale tools were developed to describe mammalian embryogenesis at the tissue, cell, genome, gene and protein levels.

At the tissue level, the platform provides a viewer of multi-omics signals across development stages in different tissues, as well as a tool for identifying new potential tissue biomarkers. At the cell level, we developed a series of data mining tools for cell clustering, gene expression pattern analysis, and potential cellular biomarker identification. At the genome level, EmAtlas offers a genome browser to display epigenetic patterns at each stage of embryonic tissues. And epigenetic modification signals in the promoter, UTR, CDS, and other regions of genes can be quantified. At the gene level, the platform can display gene multi-omics patterns, spatiotemporal biological pathways, and temporal gene expression trends. At the protein level, the platform integrated RaacFold (19) and RaacLogo (18) to explore key protein sequences, functional domains and the structure of pioneer protein factors. A self-developed analysis service, EmRanger, was proposed in the platform to provide a developmental gene set analysis system.

Taken together, we believe that EmAtlas not only provides the comprehensive timing multi-omics resource for mammalian embryonic development, but also designs a cloud platform and a series of tools for understanding the mammalian embryogenesis over multiple scales. In the future, EmAtlas will continuously update multi-omics resources and analysis strategies to provide a more detailed guideline for the development biology, regenerative medicine, assisted reproduction, and congenital disease.

## DATA AVAILABILITY

The webserver is available at http://bioinfor.imu.edu.cn/ematlas or http://ematlas.org.

No accession numbers are available.

## Supplementary Material

gkac848_Supplemental_File
