# Supplementary material for: EmAtlas: a comprehensive atlas for exploring spatiotemporal activation in mammalian embryogenesis
Source: Nucleic Acids Res. 2022 Oct 3;51(D1):D924–32. doi: 10.1093/nar/gkac848 (PMC9825456; doi:10.1093/nar/gkac848)
Supplement: gkac848_Supplemental_File [file gkac848_supplemental_file.pdf]

Supplementary materials

Pipeline of data pre-processing and analysis

The pre-processing and analysis pipeline of multi-omics data includes several analysis modules (Figure S1). Firstly, a customize script which integrated three download schemes (Aspera, Aria2 and wget) by Python was used to intelligently select the appropriate data source and download scheme according to the download speed. Once the raw data (FASTQ files) download was complete, quality control was performed by FastQC (v0.11.9, http://www.bioinformatics.babraham.ac.uk/projects/fastqc/) and quality control results for each sample were integrated through MultiQC (v1.12, https://multiqc.info/). Unqualified data were trimmed based on Trim Galore (v0.6.6, https://www.bioinformatics.babraham.ac.uk/projects/trim_galore/) to remove adaptor and low-quality reads until passing quality control. Then, filtered reads were entered into the respective omics analysis pipeline.

For transcriptome data

The filtered reads of RNA-seq were aligned to the GRCh37 human and GRCm38 mouse reference genome (NCBI) using STAR (v2.7.10a) [1], respectively. Samtools (v1.15.1) [2] was used to convert SAM files into binary BAM files. Then, the BAM files of each sample were used to quantify the expression levels by Salmon (v1.9.0) [3]. The individual quantification files were merged and normalized by the customize script. The gene identifier conversion was performed based on the gene annotation information of this study, including MGI, HGNC, UniProt, Ensemble, Entrez, etc.

For bulk RNA-seq datasets, differential expression analysis was performed by R package DEseq2 [5]. For each comparison, genes with P-value < 0.05 and Log2FC > 1 were regarded as differential expression genes (DEGs) [6]. All samples of each analysis task will undergo dimension reduction and clustering analysis. Finally, the correct analysis results will be imported into the resource database, and the error information will be returned to log files.

For single-cell RNA-seq (scRNA-seq) datasets, scanpy [7] was used to conduct differential expression analysis. This strategy will detect highly variable genes (HVGs) in different tissue types, cell clusters and unsupervised clusters. When detecting different cell clusters, the strategy first used principal component analysis (PCA) to reduce the dimension, and the detected principal components were taken as input to perform UMAP (uniform manifold approximation and projection) and t-SNE (t-distributed stochastic neighborhood embedding) analysis. These dimension reduction information will also be stored in the EmAtlas. In order to accurately identify biomarker genes in mammalian development, three ways were used to annotate candidate genes: differential genes captured by bulk RNA-seq, HVGs detected by scRNA-seq, and known biomarkers obtained by manual curation of PubMed (https://pubmed.ncbi.nlm.nih.gov/) literature (Review Genes).

For chromatin accessibility, histone modification and transcription factors binding data

This pipeline is applicable to ATAC-seq data of chromatin accessibility, ChIP-seq data of histone modifications (HMs) and transcription factors (TFs) binding data.

The filtered reads were aligned to the GRCh37 human and GRCm38 mouse reference genome (NCBI) using BWA (v0.7.17) [8]. The SAM files obtained by alignment step were converted into BAM files by Samtools (v1.15.1) [2]. Next, MACS2 (v2.2.7.1) was used to call peaks [9]. The generated BED files was converted into WIG files using Bedtools (v2.30.3) [10], and further converted into BigWig files using WigtoBigWig tool [11] for visualization in the epigenome browser tool of the EmAtlas.

After the peak signal was normalized, the pipeline can calculate three evaluation indicators of the epigenetic signals in different genomic regions (such as the promoter, untranslated region (UTR), coding sequence (CDS), first exon, etc.). These three evaluation indicators were modification signal area, single base modification value, and modification coverage percentage.

For DNA methylation data

This pipeline is applicable to bisulfite sequencing (BS-seq) of DNA methylation modifications and other omics data generated by similar sequencing technologies.

The filtered reads were aligned to the GRCh37 human and GRCm38 mouse reference genome (NCBI) using Bismark (v0.23.0) [12]. The SAM files obtained by alignment step were converted into BAM files by Samtools (v1.15.1). Then, BAM files were converted into BigWig files using Bedtools (v2.30.3) [10] and WigtoBigWig tool [11]. Finally, the normalization and

epigenetic signals calculation modules were consistent with the above process by the customize scripts.

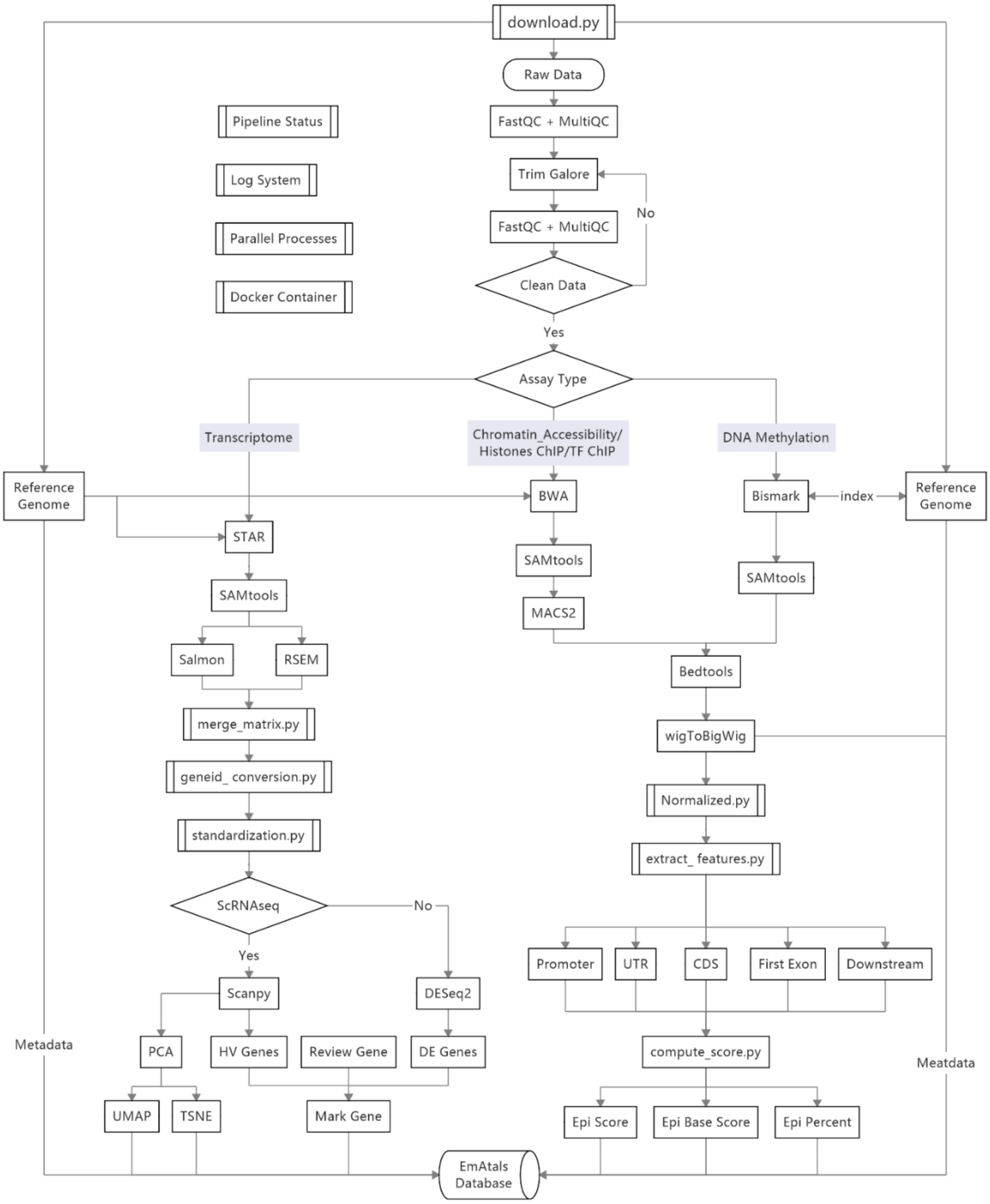

Figure S1. The workflow of multi-omics analysis management system based on Nextflow.

Reference

[1] DOBIN A, DAVIS C A, SCHLESINGER F, et al. STAR: ultrafast universal RNA-seq aligner [J]. Bioinformatics (Oxford, England), 2013, 29(1): 15-21.

[2] LI H, HANDSAKER B, WYSOKER A, et al. The Sequence Alignment/Map format and SAMtools [J]. Bioinformatics (Oxford, England), 2009, 25(16): 2078-9.

[3] PATRO R, DUGGAL G, LOVE M I, et al. Salmon provides fast and bias-aware quantification of transcript expression [J]. Nat Methods, 2017, 14(4): 417-9.

[4] LI B, DEWEY C N. RSEM: accurate transcript quantification from RNA-Seq data with or without a reference genome. BMC Bioinform 12:323 [J]. BMC bioinformatics, 2011, 12(1): 93-9.

[5] VARET H, BRILLET-GUEGUEN L, COPPEE J Y, et al. SARTools: A DESeq2- and

EdgeR-Based R Pipeline for Comprehensive Differential Analysis of RNA-Seq Data [J]. PloS one, 2016, 11(6): e0157022.
[6] LI H, LONG C, XIANG J, et al. Dppa2/4 as a trigger of signaling pathways to promote zygote genome activation by binding to CG-rich region [J]. Brief Bioinform, 2020.
[7] WOLF F A, ANGERER P, THEIS F J. SCANPY: large-scale single-cell gene expression data analysis [J]. Genome biology, 2018, 19(1): 15.
[8] LI H, DURBIN R. Fast and accurate short read alignment with Burrows-Wheeler transform [J]. Bioinformatics (Oxford, England), 2009, 25(14): 1754-60.
[9] ZHANG Y, LIU T, MEYER C A, et al. Model-based analysis of ChIP-Seq (MACS) [J]. Genome biology, 2008, 9(9): R137.
[10] QUINLAN A R. BEDTools: The Swiss-Army Tool for Genome Feature Analysis [J]. Current protocols in bioinformatics, 2014, 47: 11.2.1-34.
[11] KENT W J, ZWEIG A S, BARBER G, et al. BigWig and BigBed: enabling browsing of large distributed datasets [J]. Bioinformatics (Oxford, England), 2010, 26(17): 2204-7.
[12] KRUEGER F, ANDREWS S R. Bismark: a flexible aligner and methylation caller for Bisulfite-Seq applications [J]. Bioinformatics (Oxford, England), 2011, 27(11): 1571-2.
